# APOBEC3G induces a hypermutation gradient: purifying selection at multiple steps during HIV-1 replication results in levels of G-to-A mutations that are high in DNA, intermediate in cellular viral RNA, and low in virion RNA

**DOI:** 10.1186/1742-4690-6-16

**Published:** 2009-02-13

**Authors:** Rebecca A Russell, Michael D Moore, Wei-Shau Hu, Vinay K Pathak

**Affiliations:** 1 Viral Mutation Section, HIV Drug Resistance Program, Center for Cancer Research, National Cancer Institute at Frederick, Frederick, Maryland 21702, USA; 2 Viral Recombination Section, HIV Drug Resistance Program, Center for Cancer Research, National Cancer Institute at Frederick, Frederick, Maryland 21702, USA

## Abstract

**Background:**

Naturally occurring Vif variants that are unable to inhibit the host restriction factor APOBEC3G (A3G) have been isolated from infected individuals. A3G can potentially induce G-to-A hypermutation in these viruses, and hypermutation could contribute to genetic variation in HIV-1 populations through recombination between hypermutant and wild-type genomes. Thus, hypermutation could contribute to the generation of immune escape and drug resistant variants, but the genetic contribution of hypermutation to the viral evolutionary potential is poorly understood. In addition, the mechanisms by which these viruses persist in the host despite the presence of A3G remain unknown.

**Results:**

To address these questions, we generated a replication-competent HIV-1 Vif mutant in which the A3G-binding residues of Vif, Y^40^RHHY^44^, were substituted with five alanines. As expected, the mutant was severely defective in an A3G-expressing T cell line and exhibited a significant delay in replication kinetics. Analysis of viral DNA showed the expected high level of G-to-A hypermutation; however, we found substantially reduced levels of G-to-A hypermutation in intracellular viral RNA (cRNA), and the levels of G-to-A mutations in virion RNA (vRNA) were even further reduced. The frequencies of hypermutation in DNA, cRNA, and vRNA were 0.73%, 0.12%, and 0.05% of the nucleotides sequenced, indicating a gradient of hypermutation. Additionally, genomes containing start codon mutations and early termination codons within *gag *were isolated from the vRNA.

**Conclusion:**

These results suggest that sublethal levels of hypermutation coupled with purifying selection at multiple steps during the early phase of viral replication lead to the packaging of largely unmutated genomes, providing a mechanism by which mutant Vif variants can persist in infected individuals. The persistence of genomes containing mutated *gag *genes despite this selection pressure indicates that dual infection and complementation can result in the packaging of hypermutated genomes which, through recombination with wild-type genomes, could increase viral genetic variation and contribute to evolution.

## Background

The APOBEC3 proteins APOBEC3G (A3G) and APOBEC3F (A3F) are potent inhibitors of Vif-deficient HIV-1 [1-5]. However, in the presence of HIV-1 Vif the A3G and A3F proteins are targeted for proteasomal degradation, thereby protecting the progeny virions from their antiviral effects [6-11]. The importance of the Vif-APOBEC3 interaction in protecting HIV-1 therefore makes it a very attractive target for antiviral therapy development, as inhibiting the interaction would allow these host restriction factors to inhibit HIV-1 replication. To further elucidate the structural determinants of the Vif-APOBEC3 interaction, we and others have identified the domains of Vif that are involved in binding to A3G and A3F [12-17]. Furthermore, as a proof of principle, work by Mehle et al. has shown that Vif peptides overlapping the A3G-binding domain were able to inhibit the Vif-A3G interaction [13].

The mechanisms of action of the APOBEC3 proteins on Vif-deficient HIV-1 have been the focus of a number of studies [2,18-26] and recently reviewed in [27]. However, the effect of extensive G-to-A hypermutation on the ongoing replication of HIV-1 has not been studied in depth. Recently, Mulder et al. have shown that a replication-competent virus containing mutations in Vif residues involved in interactions with A3G displayed reduced fitness in PBMC cultures; furthermore, viral DNA in these cells contained extensive G-to-A hypermutation indicative of A3G-induced cytidine deamination [14]. In addition, among these viral clones drug-resistant variants existed that could be rescued through recombination with wild type (WT) HIV-1 following dual infection.

The mechanisms by which mutant Vif HIV-1 clones are able to maintain replication despite continued inhibition by A3G are poorly understood. To elucidate these mechanisms, we studied the growth kinetics of replication-competent HIV-1 containing the YRHHY > A5 Vif mutation in permissive CEM-SS cells and non-permissive CEM cells. We have previously shown that the YRHHY > A5 mutation renders Vif unable to efficiently bind to and inhibit A3G [15] thereby allowing us to examine the effects of A3G on replication-competent HIV-1 replication. Unlike previous work studying the presence of G-to-A hypermutation, we examined both the cellular viral and virion RNA as well as the viral DNA. The results showed that the frequency of hypermutation was highest in viral DNA, reduced in cellular viral RNA (cRNA), and lowest in virion RNA (vRNA), indicating a gradient of hypermutation. We surmise that purifying selection at multiple steps during viral replication results in the generation of this hypermutation gradient. As a consequence, viral RNAs that are unmutated or only slightly mutated are packaged in virions for the next round of infection. These observations provide an explanation for the persistence of Vif mutants defective in A3G inhibition in HIV-1 infected individuals, such as those previously reported by Simon et al [16]. We also observed complementation between replication-competent virus and virus containing stop codons in Gag, providing additional evidence that hypermutant genomes could contribute to viral variation through recombination with wild-type viral genomes [14].

## Results

### Virus containing the YRHHY > A5 mutation is inhibited in the presence of A3G and D128K-A3G but not A3F

**Figure 1:**
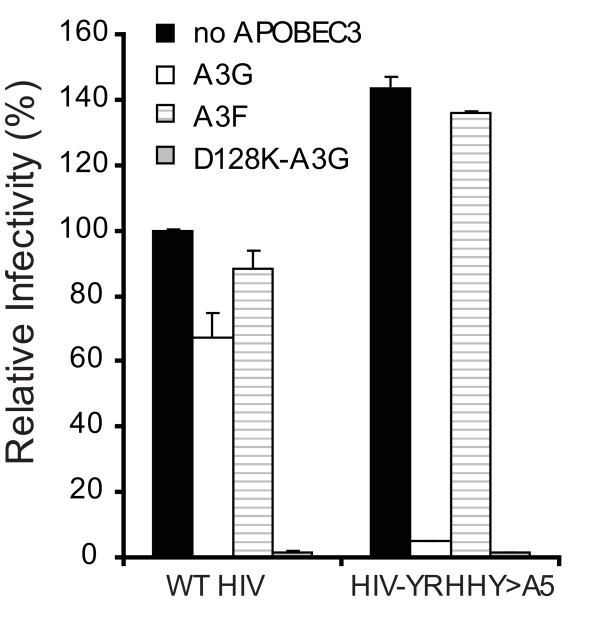
**Mutation of the YRHHY domain of Vif in the context of replication-competent HIV-1 results in loss of Vif function against A3G but not A3F**. HIV WT and pHIV-YRHHY > A5, a replication competent HIV-1 containing the YRHHY > A5 mutation, were transfected into 293T cells in the presence of A3G, A3F, or D128K-A3G (a Vif-resistant mutant of A3G). The infectivity of the virus produced from the transfected cells, harvested after 48 hours, was determined by infection of TZM-bl indicator cells and quantitation of the resulting luciferase enzyme activity. The data shown are plotted as the infectivity relative to that produced in the absence of any APOBEC3 proteins which was set to 100%, with standard deviation from two independent experiments.

Our previous studies showed that a Vif mutant (YRHHY > A5), in which the Y^40^RHHY^44 ^residues were substituted with five alanines, was unable to block the antiviral activity of A3G but was fully effective in blocking the antiviral activity of A3F [15]. To assess the effects of this Vif mutant in a multiple cycle system the YRHHY > A5 mutation was introduced into a replication-competent virus (HIV-YRHHY > A5). To confirm that HIV-YRHHY > A5 showed the expected phenotype, the mutant and HIV WT were first tested in a transient transfection system in the presence of A3G, A3F, and the D128K-A3G mutant which is resistant to HIV-1 Vif-induced degradation [15,28-31]. As expected, HIV WT was resistant to A3G and A3F but not D128K-A3G, since WT Vif can inhibit both A3G and A3F but not D128K-A3G (Fig. 1). In agreement with our previously published data [15], the HIV-YRHHY > A5 mutant virus was inhibited by A3G and D128K-A3G but not A3F.

### HIV-YRHHY > A5 is delayed in CEM cells but not CEM-SS cells

**Figure 2:**
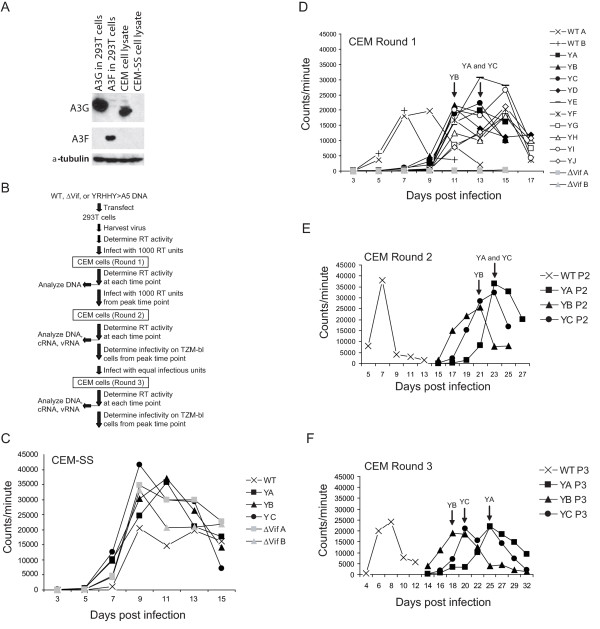
**Delayed growth kinetics displayed by HIV-YRHHY > A5 in non-permissive cells but not in permissive cells**. (A) Expression levels of A3G in CEM and CEM-SS cells. To confirm that the non-permissive CEM cells expressed A3G and the permissive CEM-SS cells did not, cell lysates were analyzed by western blotting for expression of both A3G and A3F. Expression of α-tubulin in the cell lysates was also analyzed to control for the amount of cell lysate examined. As positive controls 293T cell lysates transfected with FLAG-tagged A3G and A3F were also analyzed. (B) Schematic representation of the virus-passage protocol used. The different steps carried out at each round of infection are shown. (C) Virus growth in permissive CEM-SS cells. To determine the growth kinetics of HIV-YRHHY > A5 in permissive CEM-SS cells, 1000 RT units were added to 1 × 10^6 ^CEM-SS cells and the virus and cells were cultured at 37°C. At various time points virus-containing supernatant was removed and the RT levels were determined. As controls, HIV WT and NL4-3ΔVif were also included. The results are plotted as the scintillation counts/minute measured at each time point for 3 independent infections of HIV-YRHHY > A5 and two independent infections of HIV WT and NL4-3ΔVif. (D) Virus growth in Round 1 infection of non-permissive CEM cells. The experiment was carried out as described in FIG. 1C legend except that 10 independent infections were used for HIV-YRHHY > A5. (E) Virus growth in Round 2 infections of non-permissive CEM cells. Virus from the peak of infection of HIV-YRHHY > A5 Round 1 samples YA, YB, and YC and HIV WT was added to fresh CEM cells and passaged as described in Fig. 2C legend. (F) Virus growth in Round 3 infections of non-permissive CEM cells. Virus from the peak of infection of HIV-YRHHY > A5 Round 2 samples YA, YB and YC and HIV WT A was added to fresh CEM cells and passaged as described in FIG. 2C legend.

Next, we compared the replication characteristics of HIV-YRHHY > A5 and HIV WT in a multiple cycle assay in permissive CEM-SS cells and non-permissive CEM cells. We also used as a control, NL4-3ΔVif, which contains two stop codons resulting in the production of a truncated protein consisting of only the first 29 amino acids of Vif. To verify that the CEM cells expressed A3G and the CEM-SS cells did not, we performed western blot analysis (Fig. 2A). The results showed that the A3G protein was detectable in CEM cell lysates but not CEM-SS cells; neither the CEM nor the CEM-SS cells expressed detectable levels of A3F.

Fig. 2B shows an outline of the infection protocol used. The Round 1 input virus was produced in 293T cells and each infection was carried out with 1000 RT units of each virus and 1 × 10^6 ^CEM or CEM-SS cells. As the results in Fig. 2C show, in the permissive CEM-SS cells the RT values of HIV WT, NL4-3ΔVif (two independent infections), and HIV-YRHHY > A5 (three independent infections; curves labeled YA, YB, and YC) all peaked between days 9 and 11 and then declined, concomitant with increasing cell death. These results indicated that in the absence of A3G, HIV WT, HIV-YRHHY > A5, and NL4-3ΔVif exhibited similar replication kinetics in a spreading infection.

Next, we compared the replication kinetics of HIV WT, HIV-YRHHY > A5, and NL4-3ΔVif in the non-permissive CEM cells (Fig. 2D). HIV WT replication, as determined by RT activity, peaked at day 7 (two independent infections, labeled WT A and WT B) whereas the NL4-3ΔVif replication did not reach above background levels for the duration of the experiment (15 days; two independent infections, labeled as ΔVifA and ΔVifB); this observation indicated that in the absence of Vif, HIV-1 cannot grow in the presence of A3G. For the HIV-YRHHY > A5 mutant, ten independent infections were carried out (labeled YA through YJ); as the results in Fig. 2D show, HIV-YRHHY > A5 mutant replication peaked between days 11 and 15, indicating a 4 to 8 day delay compared to HIV WT. These results indicated that in the presence of the YRHHY > A5 mutation, which results in suboptimal Vif function, the A3G expressed in CEM cells is able to significantly delay the kinetics of HIV-1 replication. We also noted that the HIV-YRHHY > A5 viruses replicated with delayed kinetics while the NL4-3ΔVif viruses completely failed to replicate. We therefore hypothesized that the HIV-YRHHY > A5 mutant possessed a low level of Vif activity that allowed some viruses to escape the inhibitory effects of A3G, resulting in continued replication, albeit with delayed kinetics.

### No evidence of adaptive mutations in HIV-YRHHY > A5 virus passaged in CEM cells

To determine whether the HIV-YRHHY > A5 virus that replicated in CEM cells contained adaptive mutations that allowed it to inhibit A3G and thus grow in the non-permissive cells, 1000 RT unit aliquots of the HIV-YRHHY > A5 viruses from the days of peak RT for samples YA (day 13), YB (day 11), and YC (day 13) were added to fresh CEM cells (Round 2); these three samples were selected at random as they appeared to be representative of the 10 cultures that were analyzed in Fig. 2D. As the results in Fig. 2E show, the HIV-YRHHY > A5 viruses in Round 2 were further delayed, with the HIV WT (WT P2) peaking at day 7 and the mutant viruses (YA P2, YB P2, and YC P2) peaking 14 to 16 days later between days 21 and 23; the increased delay in the replication kinetics indicated that the viruses from Round 1 had not acquired any escape mutations.

We hypothesized that the increased delay seen between Rounds 1 and 2 may have been due to the fact that the RT units did not accurately reflect the level of infectious HIV-YRHHY > A5 virus present in the Round 1 peak. To test this hypothesis, 100 μl of the virus from the days of peak RT at Round 1 was added to TZM-bl cells and the level of luciferase expression measured 24 hours later. To detect luciferase expression in this system, the incoming virus must be capable of cell entry, reverse transcription, integration, and Tat expression, thus making it a more accurate reflection of infectious virus levels than the RT assay. As the results in Table 1 show, the HIV-YRHHY > A5 viruses taken from the peak RT values of Round 1 were between 7- and 8.6-fold less infectious than the HIV WT taken from the peak RT at day 7, possibly explaining the increased delay seen between Rounds 1 and 2. Based on this observation, the viruses from the days of peak RT of Round 2 were also analyzed on TZM-bl cells and, as the results in Table 1 show, equivalent volumes of the HIV-YRHHY > A5 viruses were 9.5- to 21.7-fold less infectious than the HIV WT virus. This difference was taken into consideration when setting up Round 3 infections, and equivalent amounts of infectious viruses, as quantified using the TZM-bl cells line, were added to fresh CEM cells. Surprisingly, the HIV-YRHHY > A5 viruses were delayed as much in Round 3 as they were in Round 2 with HIV WT peaking at day 8 and the HIV-YRHHY > A5 viruses peaking between days 18 and 25 (Fig. 2F). Furthermore, analysis of the Round 3 mutant viruses on TZM-bl cells showed a further drop in infectivity from 19.1- to 106.4-fold compared to HIV WT (see Table 1). The fact that the viruses from Round 2 were still delayed when added to fresh CEM cells in Round 3 further confirmed that escape mutations were not the cause of the observed virus growth.

**Table 1:** Infectivity of HIV WT and HIV-YRRHHY > A5 virus-containing supernatants from samples with peak RT activities.

Virus	Round of Infection^a^	Relative Infectivity^b^ (%) ± S.D.^c^	Fold Decrease in Infectivity
WT	1	100 ± 4.5	-
YA	1	11.6 ± 0.2	8.6
YB	1	13.6 ± 0.1	7.4
YC	1	14.2 ± 0.1	7.0

WT	2	100 ± 3.0	-
YA	2	10.6 ± 0.5	9.5
YB	2	7.3 ± 0.5	13.7
YC	2	4.6 ± 0.7	21.7

WT	3	100 ± 4.2	-
YA	3	0.9 ± 0.0	106.4
YB	3	5.2 ± 1.0	19.1
YC	3	2.2 ± 0.4	44.7

^a ^Peak time points analyzed are shown in Fig. 2.

^b ^The infectivity of equivalent volumes of cell supernatants was assessed using TZM-bl cells. Luciferase activities in infected cell lysates were measured 24 hours after infection.

^c ^S.D., standard deviation.

### HIV-YRHHY > A5 viral DNA, cRNA, and vRNA exhibit a gradient of hypermutation after replication in CEM cells

**Figure 3:**
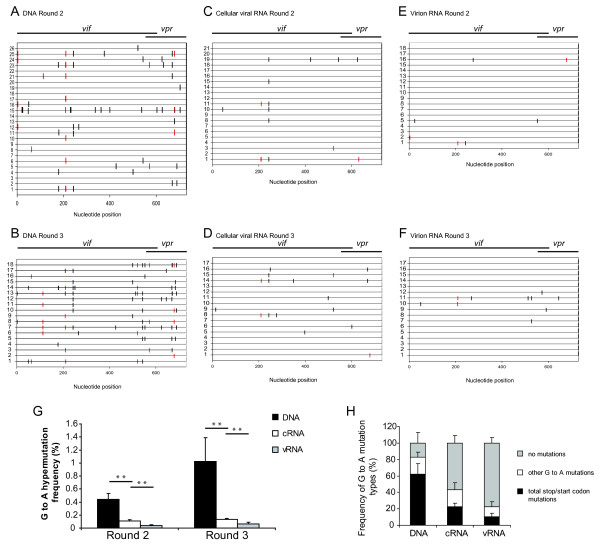
**Gradient of A3G-induced hypermutation across proviral DNA, cellular viral RNA (cRNA), and virion RNA (vRNA) observed in the *vif *of HIV-YRHHY > A5**. (A and B) Schematic representation of a sample of proviral DNA sequences of individual clones from Rounds 2 and 3. Genomic DNA was extracted from infected CEM cells at the peak of infection (as determined by RT activity). A 730 bp region including the *vif *gene and a portion of the *vpr *gene was amplified, cloned, and sequenced. Each horizontal line represents an individual clone. Each vertical line represents a G-to-A mutation. Red vertical lines represent G-to-A mutations that would result in a loss of Vif production due to either mutation of the start codon or insertion of a premature stop codon. Red vertical lines in the Vif/Vpr overlapping region are mutations that altered the Vpr start codon or generated stop codons in the Vif or Vpr open reading frames. Some vertical lines appear to be thick because two or more thin lines are very close to each other. (C and D) Schematic representation of a sample of cRNA sequences of individual clones from Rounds 2 and 3. The layout is as described above except that each clone originates from cRNA extracted from infected CEM cells at the peak of virus infection. (E and F) Schematic representation of a sample of vRNA sequences of individual clones from Rounds 2 and 3. The layout is as described above except that each clone originates from vRNA extracted from virus-containing supernatant at the peak of virus infection. (G) Graphical representation of the G-to-A hypermutation frequency from each round of infection. The frequency of G-to-A hypermutation in the proviral DNA, cRNA, and vRNA across each individual infection (YA, YB and YC) for Rounds 2 and 3 was determined. Statistical significance was calculated using the t-test assuming equal variance with a one-tailed analysis. (H) Graphical representation of the type of G-to-A mutations observed in each individual clone in the proviral DNA, the cRNA, and the vRNA. The sequences from Rounds 2 and 3 were separated into 3 different groups – those that had G-to-A mutations that would destroy expression of either Vif, Vpr, or both; those that had G-to-A mutations that did not destroy protein production and those that had no G-to-A mutations within the region sequenced. For the proviral DNA 139 sequences were analyzed, for the cRNA 108 sequences were analyzed, and for the vRNA 127 sequences were analyzed.

The observation that the HIV-YRHHY > A5 virus that replicated with delayed kinetics was still delayed when added to fresh CEM cells at equivalent levels of infectious units, suggested the absence of adaptive mutations. Furthermore, sequence analysis of *vif *from individual clones of Rounds 1, 2, and 3 did not show any consensus mutations indicative of escape mutants (data not shown). We hypothesized that because the YRHHY > A5 mutant possessed a low level of Vif activity, this allowed some viruses to escape the inhibitory effects of A3G, resulting in continued replication with delayed kinetics. To test this hypothesis, we first sequenced viral DNA from Rounds 2 and 3 to determine whether any of the proviruses lacked G-to-A hypermutation indicative of A3G-mediated inhibition. Cellular DNAs were extracted, a 730-bp region spanning the *vif *gene and a portion of the *vpr *gene was amplified, cloned, and individual clones were sequenced. The results in Fig. 3A and 3B show a representative set of sequences obtained from Rounds 2 and 3, respectively, with the horizontal lines depicting individual clones and the vertical lines indicating G-to-A mutations; red vertical lines represent G-to-A mutations that would result in either a loss of expression due to mutation of the start codon or a truncated protein due to the formation of an early termination codon. In addition to the G-to-A mutations, the viral DNAs also had other mutations at a frequency that was 11.4-fold lower than the G-to-A mutations (0.06% per nucleotide sequenced; data not shown). The mutation frequency of non G-to-A changes was not altered between HIV WT and HIV-RHHY > A5. The results showed that most viral DNAs had extensive G-to-A hypermutation; 69 and 70 viral DNAs were sequenced from Rounds 2 and 3, respectively; the G-to-A mutation frequencies for Round 2 and 3 were 0.44% and 1.02% per nucleotide sequenced, respectively. In agreement with previously published data, the G-to-A mutations predominantly occurred in GG dinucleotides, in which the 5' G was mutated to A (Table 2) [19,32-35]. For the 139 viral DNA clones sequenced, the overall G-to-A mutation frequency was 0.70% per nucleotide sequenced. The mutation frequency in viral DNAs from Rounds 2 and 3 was significantly higher than the 0.02% mutation frequency (4 mutations in 23 sequences) observed in viral DNAs analyzed from HIV WT infections (*P *< 10^-6^). An average of 5.12 G-to-A mutations were observed per 730 nucleotides of sequence from the Vif/Vpr region analyzed. Assuming a Poisson distribution, we expected only 0.5% of the 139 sequences analyzed to have no G-to-A substitutions. However, we observed that 26 of the 139 (18%) sequences lacked any G-to-A mutations. This analysis supported our hypothesis and suggested that these viruses escaped A3G-mediated inhibition.

**Table 2:** Dinucleotide context of G-to-A mutations in Vif/Vpr and DIS/Gag regions.

Virus (Region sequenced)	Dinucleotide context of G-to-A Mutations				
	
	GG^a ^(%)	GA (%)	GC (%)	GT (%)	Total
WT (Vif/Vpr)					
DNA	1 (25%)	3 (75%)	0	0	4
Cellular viral RNA	0	0	0	0	0
Virion RNA	7 (32%)	11 (50%)	1 (5%)	3 (14%)	22
HIV-YRHHY>A5 (Vif/Vpr)					
DNA	620 (87%)	83 (12%)	6 (1%)	3 (0.4%)	712
Cellular viral RNA	81 (86%)	9 (10%)	3 (3.2%)	1 (1%)	94
Virion RNA	34 (69%)	10 (20%)	3 (6%)	2 (4%)	49
HIV-YRHHY>A5 (DIS/Gag)					
DNA	54 (87%)	8 (13%)	0	0	62
Cellular viral RNA	74 (89%)	3 (4%)	3 (4%)	3 (4%)	83
Virion RNA	20 (74%)	4 (15%)	0	3 (11%)	27

^a ^The first G nucleotide in the GG dinucleotide is the target of G-to-A mutation.

Our hypothesis predicted that only viral genomes that had escaped A3G-mediated inhibition and hypermutation would be present in viral RNA. To test this hypothesis, we isolated cRNAs and vRNAs and obtained sequences of clones generated from cDNAs. Representative results obtained from Rounds 2 and 3 for cRNA-derived cDNAs are shown in Figs. 3C and 3D, respectively, and the results for vRNA-derived cDNAs are shown in Figs. 3E and 3F, respectively. The analysis showed that the frequency of clones that did not have any G-to-A mutations was increased from 18% to 57% in cRNAs; the frequency of clones without any G-to-A mutations was further increased to 77% in vRNAs. The overall frequency of G-to-A mutations in cRNAs and vRNAs was reduced to 0.12% and 0.05% for total nucleotides sequenced, respectively (Fig. 3G). In agreement with previously published data, the G-to-A mutations predominantly occurred in GG dinucleotides, in which the 5' G was mutated to A (Table 2) [19,32-35]. The G-to-A mutation frequency of all the *vif *and *vpr *sequence data obtained from the viral DNA, cRNA, and vRNA from each infection (YA, YB and YC) at Rounds 2 and 3 are shown in Fig. 3G and Table 3. A total of 139 sequences from viral DNA (101,470 nucleotides), 108 sequences from cRNA (78,840 nucleotides), and 127 sequences from vRNA (92,710 nucleotides) were analyzed. The differences in the G-to-A mutation frequency between viral DNA and cRNA were highly significant (*P *= 0.0038 and *P *= 0.0139 for Rounds 2 and 3, respectively; Student's *t*-test). Similarly, the differences in the hypermutation frequency between cRNA and vRNA were also highly significant (*P *= 0.0074 and *P *= 0.0089 for Rounds 2 and 3, respectively). These observations establish that there is a gradient of hypermutation, with the frequency of G-to-A mutations being the highest in viral DNA, intermediate in cRNA, and lowest in vRNA.

**Table 3:** Analysis of mutations in the Vif/Vpr and DIS/Gag regions.

	Vif/Vpr Region				DIS/Gag Region	
	HIV-1 WT		HIV-YRHHY>A5		HIV-YRHHY>A5	
	
	G-to-A Mutations/ Total G nts^a^	Other Mutations/ Total nts^b^	G-to-A Mutations/Total G nts	Other Mutations/ Total nts	G-to-A Mutations/ Total G nts^c^	Other Mutations/ Total nts^d^

DNA	4/3910	0/16,790	712/23,630	66/101,470	62/2856	14/9000
Cellular Viral RNA	0/2550	3/10,950	94/18,360	43/78,840	83/13,804	37/43,500
Virion RNA	22/12,580	14/54,020	49/21,590	82/92,710	27/11,424	15/36,000

^a ^Total G nucleotides (nts) in the Vif/Vpr region were 170 per sequence.

^b ^Total nucleotides in the Vif/Vpr region were 730 per sequence.

^c ^Total G nucleotides in the DIS/Gag region were 119 per sequence.

^d ^Total nucleotides in the DIS/Gag region were 375 per nucleotide.

We also determined the frequency of G-to-A mutations present in vRNA obtained from HIV WT virus infections. We found 22 G-to-A mutations in 74 sequences (54,020 nucleotides), providing a mutation frequency of 0.04%; unlike the G-to-A mutations observed in the HIV-YRHHY > A5 samples, the mutations did not predominantly occur in the GG dinucleotide context (Table 2). The G-to-A mutation frequency in Rounds 2 and 3 vRNAs obtained from HIV-YRHHY > A5 (0.05%) was not significantly different from that observed for HIV WT vRNAs (*P *= 0.5535).

An in-depth analysis of the G-to-A mutations was performed to analyze the impact of the mutations on *vif *and *vpr *gene products (Fig. 3H and Table 4). A high proportion of the viral DNA clones (60%) had G-to-A mutations that resulted in the formation of early termination codons or mutation of the start codon; the frequency of these mutations that would result in the loss of a functional Vif or Vpr protein was reduced to 22% and 10% in cRNA and vRNA, respectively (*P *= 1.43 × 10^-5 ^and *P *= 2.97 × 10^-4^; Student's *t *test). In contrast, the frequency of clones with no G-to-A mutations was 18% in viral DNA, and increased to 57% and 77% in cRNAs and vRNAs, respectively. Although we do not expect the loss of Vif or Vpr proteins to affect transcription of the viral DNA, it is likely that some G-to-A mutations would result in the loss of the viral transcriptional activator Tat protein, or that some G-to-A mutations would occur in the viral promoter regions, interfering with transcription. These observations strongly suggest that purifying selection pressure results in proviruses with no mutations (or those with fewer detrimental G-to-A mutations) being transcribed into cellular RNA.

**Table 4:** Vif/Vpr and Gag sequences containing G-to-A mutations that resulted in Stop/Start codon mutations, other mutations, or no mutations.

Sample	Total Sequences with Stop/Start Codon Mutations (%)	Total Sequences w/ Other Mutations (%)	Total Sequences w/ No Mutations (%)
Vif/Vpr			
DNA (Round 2 +3)	83 (59.7%)	30 (21.6%)	26 (18.2%)
Cellular Viral RNA (Round 2+3)	24 (22.2%)	22 (20.4%)	62 (57.4%)
Virion RNA (Round 2+3)	13 (10.2%)	16 (12.6%)	98 (77.2%)
Gag			
DNA (Round 2)	17 (70.8%)	2 (8.3%)	5 (20.8%)
Cellular Viral RNA (Round 2)	26 (22.4%)	24 (20.7%)	66 (56.9%)
Virion RNA (Round 2)	6 (6.3%)	12 (12.5%)	78 (81.3%)

**Figure 4:**
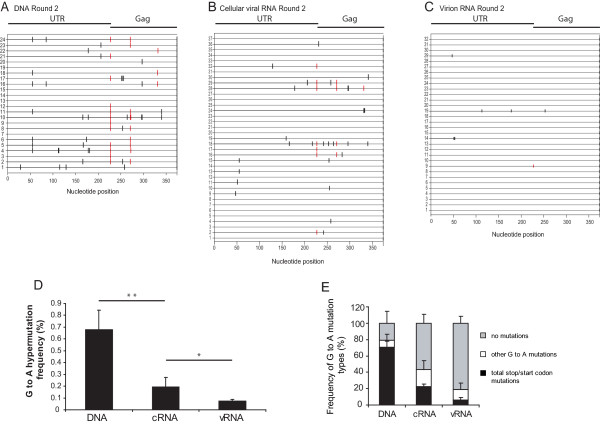
**Gradient of A3G-induced hypermutation across proviral DNA, cellular viral RNA (cRNA), and virion RNA (vRNA) observed in the untranslated leader region (UTR) and the beginning of *gag *of HIV-YRHHY > A5**. (A) Schematic representation of a sample of proviral DNA sequences of individual clones from Round 2. (B) Schematic representation of a sample of cRNA sequences of individual clones from Round 2. (C) Schematic representation of a sample of vRNA sequences of individual clones from Round 2. Samples were extracted as described in FIG. 3A–F legend. (D) Graphical representation of the G-to-A hypermutation frequency from Round 2 of infection. The frequency of G-to-A hypermutation in the proviral DNA, cRNA, and vRNA across each individual infection (YA, YB and YC) for Round 2 was determined and presented as described in FIG. 3G legend. (E) Graphical representation of the type of G-to-A mutations observed in each individual clone in the proviral DNA, the cRNA, and the vRNA. The analysis was carried out as described in FIG. 3H legend. For the proviral DNA, 24 sequences were analyzed, for the cRNA 116 sequences were analyzed, and for the vRNA 96 sequences were analyzed.

We considered two possible explanations for the reduction in G-to-A mutations observed in vRNA compared to cRNA. Firstly, we hypothesized that G-to-A mutations in the viral packaging sequence and/or dimer initiation site (DIS) would prevent the packaging of extensively hypermutated RNAs. However, analysis of the 5' untranslated region did not reveal the presence of a high number of G-to-A mutations in these regions; only 1 G-to-A mutation was found in the DIS region and that was in the cRNA and a total of 6 mutations were found in the packaging sequence (2 in each of the DNA [2 out of 24], cRNA [2 out of 116] and vRNA [2 out of 96]). Furthermore, there did not appear to be a gradient of hypermutation between the cellular and viral RNA suggesting that this area is not under selection pressure, although the numbers of mutations in this region are too small to draw definitive conclusions. Secondly, we hypothesized that inactivating mutations in HIV-1 *gag *would result in the loss of functional proteins that are essential for virus production. To test these hypotheses, we carried out sequencing analysis of the viral untranslated leader and the beginning of the *gag *gene. Representative results obtained from viral DNAs, cRNA, and vRNA from Round 2 are shown in Fig. 4A, B, and 4C, respectively. The frequencies of G-to-A mutations are summarized in Fig. 4D and Table 3; 24 sequences (9,000 nucleotides) were analyzed from proviral DNA, 116 sequences (43,500 nucleotides) were analyzed from cRNA, and 96 sequences (36,000 nucleotides) were analyzed from vRNA. In agreement with the results obtained with sequences acquired from the *vif*/*vpr *genes, there was a gradient of G-to-A mutations, with the highest G-to-A mutation frequencies in viral DNA (0.68%), intermediate mutation frequencies in cRNA (0.19%), and the lowest mutation frequencies in vRNA (0.08%). Furthermore, in agreement with previously published data, the dinucleotide context of the G-to A-changes was predominantly GG (Table 2) [19,32-35].

A more detailed analysis of the G-to-A mutations is shown in Fig. 4E and Table 4. The frequency of clones with no G-to-A mutations was approximately 21% in viral DNAs, which was increased to approximately 57% and 81% in cRNAs and vRNAs, respectively. The differences in the G-to-A mutation frequencies between viral DNA and cRNA were significant (*P *= 0.004), as were differences between cRNA and vRNA (*P *= 0.008). The frequency of G-to-A mutations that inactivated the *gag *gene by generating premature stop codons or mutating the start codon was 71% in the viral DNA, and was decreased to 22% and 6% in cRNA and vRNA, respectively. These results indicated that purifying selection pressure was operating against genomes that had inactivating mutations in the *gag *gene. The observation that a few of the viral RNA-derived sequences had inactivating mutations in the *gag *gene strongly indicated that these genomes were packaged by co-infection of the virus producing cell with another virus and complementation.

## Discussion

To overcome the effects of the antiviral A3G protein, the HIV-1 Vif protein binds to A3G and targets it for degradation using the cellular proteasomal degradation pathway [6-11]. However, in some infected individuals, HIV-1 variants with Vif mutations that inhibit the Vif-A3G interaction have been identified [16]. In these individuals, it is unclear how the Vif variants persist in the population since they are expected to be inhibited by the A3G protein. The work described here presents mechanisms by which these Vif variants may survive in the population by showing, for the first time, that a gradient of hypermutation exists for the integrated proviral DNA, the cellular viral RNA, and the virion RNA. Based on these observations, we hypothesize that purifying selection is occurring at each stage of virus production, including transcription, mRNA stability, nuclear-cytoplasmic transport, translation, and virion assembly. The integrated genomes with extensive hypermutation may not be transcribed, possibly due to mutations in the promoter regions or in the tat gene, thereby preventing the extensively hypermutated genomes from contributing to the gene pool of the viral population. Mutations in the transcribed RNA may reduce their stability and they may be degraded before they can be translated; for example, the RNAs may be rapidly degraded through a nonsense-mediated RNA decay mechanism due to the generation of premature stop codons [36]. Additionally, in the absence of co-infection with a wild-type virus, transcribed genomes encoding *gag *genes with early termination codons or mutated start codons will not be able to assemble virus particles, thereby allowing only unmutated genomes or minimally mutated genomes to both produce, and be packaged into, progeny virions. Despite this purifying selection at multiple steps, we were able to detect viral genomes containing stop codons in *gag*; the presence of these genomes in vRNA indicates dual infection and complementation of the *gag *defect. Thus, hypermutated genomes can be packaged in viral particles, and the G-to-A mutations could contribute to viral variation through recombination. Recombination allowing drug resistance mutations to jump from 'dead' hypermutated genomes to WT HIV-1 has recently been observed by Mulder et al [14]. The frequency of G-to-A mutations in vRNAs derived from Vif-defective HIV-1 was not significantly different from the vRNAs derived from HIV WT even after 61 days in culture, suggesting that hypermutation does not increase, or only moderately increases, the overall mutation rate of the replicating viral population. The strong purifying selection and the significantly reduced levels of G-to-A mutations in the vRNA observed in this study reduces the probability of hypermutation contributing to viral variation; however, the extent to which hypermutated genomes, packaged by complementation, undergo recombination with wild-type genomes during the course of natural HIV-1 infection, is not known.

We observed that 18% of the viral DNAs did not have G-to-A mutations in the 730 nucleotide Vif/Vpr region sequenced. It is possible that these viral DNAs contained mutations in the approximately 9000 nucleotides of their genome that we did not sequence. It is also possible that a proportion of the 18% of the viral DNAs without G-to-A mutations did not package A3G, and as a result escaped G-to-A hypermutation. We observed that the virions produced in Round 3 had an average infectivity of 2.76% of wild-type virus, suggesting that a small proportion of the virions either had no mutations or had few mutations that did not prevent virus production, infection, and expression of the Tat protein. One possible mechanism to explain how these viruses retained their infectivity is the low or absent levels of A3G expression in a subset of the CEM cells, leading to production of virions that do not contain A3G. Another possible mechanism is that a small percentage of virions are produced from A3G-expressing cells but did not package A3G and thereby escaped inhibition. We previously estimated that 7 ± 4 A3G molecules are packaged in virions [37]; if we assume a Poisson distribution, we estimate that only 0.09% of the virions would fail to package A3G. We have also observed that A3G inhibits viral DNA synthesis and integration, and the efficiency of integration in the presence of A3G is only about 3% [24]. Thus, in the integrated viral DNA pool, the frequency of non-mutated viral genomes would increase from 0.09% to 3.0%. This predicted frequency of non-mutated genomes is close to the 2.76% infectivity of the Round 3 virions; however, additional experiments are needed to verify the hypothesis that some virions retain infectivity because they do not package A3G and therefore are not subjected to hypermutation.

Interestingly, we were unable to detect the presence of any Vif-based escape variants despite a total of up to 61 days in culture. This observation does not exclude the possibility that mutations elsewhere in the genome compensated for the defects in Vif, resulting in restoration of the replicative capacity as recently observed by Hache et al [38]. However, the fact that the mutant Vif virus continued to show delayed growth kinetics, and indeed was more delayed with each round of replication, argues against the presence of any escape variants in our experiments.

The observed reductions in the frequencies of G-to-A mutations in the Vif/Vpr region could be the result of either direct or indirect purifying selection. The reductions in the frequencies of G-to-A mutations in the cRNAs are most likely due to mutations elsewhere in the genome that affect transcription, mRNA stability, and mRNA transport. The purifying selection against these mutations could indirectly reduce the frequency of mutations in the Vif/Vpr region by selecting for viral genomes with lower levels of hypermutation. On the other hand, the HIV-YRHHY > A5 mutant possessed some Vif function since it replicated with delayed kinetics while the NL4-3ΔVif mutant failed to replicate. Thus, there could be direct purifying selection against more deleterious mutations in Vif.

Finally, the observation that the HIV-YRHHY > A5 mutant exhibited a significant delay in replication kinetics for over two months, with no evidence of adaptive mutations, suggests that the Vif-A3G interaction could be a promising target for antiviral drug development.

## Conclusion

These results show for the first time that HIV-1 genomes that have been hypermutated by APOBEC3 proteins are subjected to purifying selection at multiple steps during viral replication, including transcription, mRNA stability, mRNA transport, and virus production. As a result of this purifying selection, a gradient of hypermutation exists, with the viral DNAs containing the highest levels of mutations, cellular viral RNAs containing intermediate levels of mutations, and viral RNAs containing low levels of mutations. The frequency of G-to-A mutations in vRNAs derived from Vif-deficient HIV-1 was not significantly different from the vRNAs derived from HIV WT even after 61 days in culture, suggesting that hypermutation does not increase, or only moderately increases, the overall mutation rate of the replicating viral population.

## Methods

### Plasmid construction and cell culture

The YRHHY > A5 mutation that renders HIV-1 Vif unable to efficiently bind to A3G was inserted into the replication-competent HIV-1 plasmid pNL4-3 [39] using overlapping PCR to generate pHIV-YRHHY > A5. The forward primer VifF, 5'CAGGGAGATTCTAAAAG3', and the reverse primer YRHHYmutR, 5'CTTATTTTTGGATTAGTACTTTCAGCGGCAGCTGCAGCAAACCAGTCCTTAGCTTTCC3', were used to amplify the N-terminal region of Vif. The C-terminal portion of Vif was amplified using the forward primer YRHHYmutF, 5'GGAAAGCTAAGGACTGGTTTGCTGCAGCTGCCGCTGAAAGTACTAATCCAAAAATAAG3', and the reverse primer VifR, 5'GGATAAACAGCAGTTGTTGC3'. The resulting amplicons were then combined in a second round PCR using the primers VifF and VifR. The final product was digested with AgeI plus EcoRI and cloned into AgeI plus EcoRI digested pNL4-3, displacing the WT Vif and replacing it with Vif containing the YRHHY > A5 mutation to create pHIV-YRHHY > A5.

The modified human embryonic kidney cell line, 293T [40] and the HeLa-derived HIV-1 reporter cell line, TZM-bl [41,42], which encodes the firefly luciferase gene under the control of the HIV-1 Tat-responsive promoter, were maintained in complete medium (CM) which consisted of Dulbecco's modified Eagle's medium (DMEM) supplemented with 10% fetal calf serum, 1% penicillin/streptomycin, and 1% glutamine. The lymphoid cells CEM and CEM-SS [43,44] were maintained in CEM-CM which consisted of RPMI supplemented with 10% fetal calf serum, 1% penicillin/streptomycin, and 1% glutamine.

### Virus production and titration

For virus production, 293T cells, seeded at 4 × 10^6 ^per 100-mm diameter dish were transfected using polyethylenimine (PEI; 25 kDa, Sigma) with modification of a previously described procedure [45]. For each transfection, 20 μg of either HIV WT or pHIV-YRHHY > A5 were cotransfected with 1.2 μg pGL, which expresses the green fluorescent protein from a cytomegalovirus immediate early promoter (Invitrogen); the proportion of GFP-positive cells was determined to estimate the transfection efficiency. The virus-containing supernatant was harvested 48 hours after transfection, filtered through a 0.45 μm filter, and diluted in CM. TZM-bl cells were seeded at 4 × 10^3 ^cells per well in white flat-bottomed 96-well plates, and 24 hours later infected with virus supernatant containing 5 ng of p24 capsid protein, as determined using the p24 ELISA kit (Perkin Elmer). Another 24 hours later, the culture medium was removed and replaced with 100 μl of CM without phenol red, and 100 μl of britelite luciferase solution (Perkin Elmer). After 1 minute incubation, the level of luciferase activity was measured using a LUMIstar Galaxy luminometer. Virus made by infection of CEM cells was added undiluted to the TZM-bl cells.

To determine whether the YRHHY > A5 Vif mutation displayed the expected phenotype in the presence of the different APOBEC3 proteins, 293T cells, seeded at 8 × 10^5 ^cells per well of a 6-well plate, were transfected using PEI with 6 μg of either pNL4-3 or pHIV-YRHHY > A5 and 0.5 μg of either A3G [46,47], A3F [1] or the D128K-A3G mutant [31]. At 48 hours post-transfection, the virus-containing supernatant was harvested and filtered through a 0.45 μm filter. The virus titers were then determined using TZM-bl cells as described above.

### RT assay

To determine the RT activity of virus made by transient transfection, 20 μl of virus-containing supernatant were analyzed using the Quan-T-RT assay system (Amersham). The samples were then analyzed using the 1600 TR Liquid Scintillation Analyzer (Packard). To determine the RT activity of virus made by infection of CEM cells, 1 ml of virus-containing supernatant was centrifuged at 82,000 × g for 1 hour to pellet the virus. The supernatant was removed and the virus pellet resuspended in 40 μl of phosphate buffered saline before being analyzed as described above.

### CEM and CEM-SS cell infection

CEM and CEM-SS cells were seeded at 1 × 10^6 ^cells in 1 ml CEM-CM in 25 cm^3 ^flasks and combined with an aliquot of virus that corresponded to 1000 scintillation counts/minute (referred to in the remainder of the text as 1000 RT units) in a final volume of 200 μl CEM-CM on day one of infection. The virus-cell solution was incubated at 37°C with 5% CO_2 _for 5 hours, after which an additional 5 ml CEM-CM was added. At two day intervals (days 3, 5, 7 etc. post-infection), the virus and cell suspension was mixed by pipetting, and 4 ml of cells and virus-containing supernatant was removed and centrifuged at 400 × g for 3 minutes. The virus-containing supernatant was then removed and filtered through a 0.45 μm filter and a 1 ml aliquot was stored at -70°C for RT assays. The remaining supernatant was stored at -70°C for reinfection. The virus-infected cells were resuspended in 300 μl of PBS and stored at -70°C for DNA and RNA extraction. A 4 ml aliquot of fresh CEM-CM was then added to the remaining 2 ml cell and virus suspension and the sample incubated for another 2 days.

### DNA extraction and PCR

DNA was extracted from 1 × 10^6 ^virus-infected cells using the FlexiGene DNA kit (Qiagen) and resuspended in 100 μl of buffer (FG3). A 2 μl aliquot of the extracted DNA was then used in a PCR reaction with 1 μl High Fidelity Platinum Taq (Invitrogen) and 20 pmoles each of the forward and reverse primers. The primers VifF and VifR were used to amplify the Vif gene. The dimer initiation site and beginning of *gag *was amplified using the primers DIS-F (5'GTCTGTTGTGTGACTCTGGTAAC3') and DIS-R (5'CCTGTCTGAAGGGATGGTTGTAG3').

### RNA extraction, DNase treatment, and RT-PCR

Viral RNA was extracted using the QIAamp viral RNA mini kit (Qiagen). Briefly, a 140 μl aliquot of unconcentrated virus at the peak of infection (as determined using the RT assay) was combined with 560 μl Buffer AVL containing carrier RNA and the extracted RNA was eluted from the column in 60 μl of Buffer AVE. A 25 μl aliquot of the extracted RNA was then combined with 1 μl Turbo DNase (Ambion), 5 μl 10× Buffer and 19 μl RNase-free dH_2_O. The DNase digestion was performed at 37°C for 30 minutes, after which 5 μl Inactivation reagent (Ambion) was added and incubated at room temperature for 2 minutes with regular mixing. The Inactivation reagent was removed by centrifugation at 10,000 × g for 2 minutes and a 2 μl aliquot of the DNase-treated RNA was amplified in an RT-PCR reaction using Superscript III One-step RT-PCR mix (Invitrogen). Briefly, the DNase-treated RNA was combined with 25 μl 2× Buffer, 1 μl superscript III RT-Taq mix, 20 μl RNase-free dH_2_O and 10 pmoles each of the forward and reverse primers. To amplify the Vif gene, the forward primer NL43-seq-3911F (5'GCAGGATATGTAACTGACAG3') and the reverse primer VifR were used. To amplify the dimer initiation site and beginning of *gag*, the primers DIS-F and DIS-R were used. As a control for the efficiency of the DNase treatment, each reaction was also set up with High Fidelity Platinum Taq without RT.

Cellular RNA was extracted from 1 × 10^6 ^virus-infected cells using the RNAqueous-4PCR kit (Ambion) and eluted from the column in 50 μl of Elution solution. A 25 μl aliquot of the extracted RNA was then DNase-treated and used in an RT-PCR reaction as described above.

### Cloning of PCR products in TA vectors

Following PCR or RT-PCR, the resulting PCR amplicons were resolved on a 1% agarose gel, the relevant products were extracted using the PureLink Quick gel extraction kit (Invitrogen), and eluted in 50 μl TE Buffer prewarmed to 65°C. The eluted PCR product was then used in the TOPO TA cloning reaction (Invitrogen). The resulting white colonies were grown in Luria broth and the plasmid DNA extracted using the QIAprep Turbo kit (Qiagen). The individual clones were then sequenced; for Vif sequencing, the primer NL43-seq-4921F (5'GAGATCCAGTTTGGAAAGGAC3') was used; for sequencing of the dimer initiation site and the beginning of *gag*, the primer DIS-R was used.

### Western blot for detection of endogenous A3G and A3F

An aliquot of 2 × 10^7 ^CEM and CEM-SS cells were lysed in 500 μl of lysis buffer (50 mM Tris-HCl, pH 7.4 with 150 mM NaCl, 1 mM EDTA and 1% Triton X-100), containing Protease Inhibitor Cocktail (Roche), by incubation with gentle agitation for 10 min. The cellular debris was removed by centrifugation at 10,000 × g for 10 min. The cell lysates were then analyzed by polyacrylamide gel electrophoresis and western blotting. For detection of A3G, the rabbit anti-A3G antiserum ApoC17 [48,49] at a dilution of 1:5,000 was used, followed by a horseradish peroxidase (HRP)-labeled goat anti-rabbit secondary antibody (Sigma) at a 1:10,000 dilution; for detection of A3F, a rabbit anti-human A3F antibody (Immunodiagnostics) at a dilution of 1:5,000 was used, followed by the same secondary antibody as above at a dilution of 1:10,000. As a control for the amount of total protein, α-tubulin was detected using mouse anti-α-tubulin antibody (Sigma) at a 1:5,000 dilution, followed by an HRP-labeled goat anti-mouse secondary antibody (Sigma) at a 1:10,000 dilution. The proteins were visualized using the Western Lighting Chemiluminescence Reagent Plus kit from PerkinElmer. As positive controls, 293T cell lysates containing N-terminally FLAG-tagged A3G and A3F were analyzed.

## Abbreviations

HIV-1: human immunodeficiency virus type 1; Vif: viral infectivity factor; APOBEC3G and A3G: apolipoprotein B mRNA-editing enzyme catalytic polypeptide-like 3G; APOBEC3F and A3F: apolipoprotein B mRNA-editing enzyme catalytic polypeptide-like 3F.

## Competing interests

The authors declare that they have no competing interests.

## Authors' contributions

RAR performed all experiments. VKP and RAR designed the studies and carried out data analysis. MDM and WSH provided valuable intellectual input in the design and analysis of the experiments. VKP supervised and directed the studies and data analysis. All authors approved and contributed to the preparation of the final manuscript.
